# A novel method for the measurement of hepatitis C virus infectious titres using the IncuCyte ZOOM and its application to antiviral screening

**DOI:** 10.1016/j.jviromet.2015.03.009

**Published:** 2015-06-15

**Authors:** Hazel Stewart, Christopher Bartlett, Douglas Ross-Thriepland, Joseph Shaw, Stephen Griffin, Mark Harris

**Affiliations:** a School of Molecular and Cellular Biology, Faculty of Biological Sciences, University of Leeds, Leeds LS2 9JT, United Kingdom; b Leeds Institute of Cancer and Pathology, Faculty of Medicine and Health, St James's University Hospital, University of Leeds, Leeds LS9 7TF, United Kingdom

**Keywords:** Hepatitis C virus, Titration, IncuCyte ZOOM, Diagnosis, Antivirals

## Abstract

Hepatitis C virus (HCV) is a significant human pathogen infecting 3% of the world population. An infectious molecular clone capable of replicating and releasing infectious virions in cell culture has only been available since 2005, leaving a significant knowledge gap concerning post-RNA replication events such as particle assembly, trafficking and release. Thus, a fast, efficient and accurate method of measuring infectious viral titres is highly desirable. Current methods rely upon manual counting of infected cell foci and so are both labour-intensive and susceptible to human error. Here, we report a novel protocol, which utilises the IncuCyte ZOOM instrument and related software to accurately count infected cells and extrapolation of this data to produce an infectious titre, reported as infectious units per millilitre (IU/mL). This method reduces cost, time and error in experiments. We also demonstrate that this approach is amenable to high-throughput compound screening, thereby expediting the identification of novel antivirals.

## Highlights

• Hepatitis C virus (HCV) is a significant human pathogen, causing severe liver disease.
• Accurate quantification of viral titres is essential for the majority of assays.
• The current methods of HCV titration and quantification are laborious and imprecise.
• We report a novel method for calculating infectious HCV titres using the IncuCyte ZOOM.
• This method has applications for screening of novel antiviral compounds.

## 1 Introduction

Hepatitis C virus (HCV) is estimated to infect approximately 170 million people worldwide (Mohd Hanafiah et al., 2013) and is the major causative agent of non-alcoholic liver disease, hepatocellular carcinoma and liver transplants in the developed world. Although potent direct-acting antivirals have recently become available, the high cost of treatment and an unpredictable level of viral resistance, which may emerge following therapy on a wide scale, indicate that this virus will continue to exert a significant health and economic burden for the foreseeable future. Additionally, basic research upon HCV remains a priority as the primary focus gradually shifts towards vaccine development.

Until 2005, HCV research was significantly hampered by an inability to recapitulate the full infectious cycle of the virus in cell culture systems. However, identification and characterisation of the genotype 2a JFH-1 strain, which both efficiently replicates its genome and releases infectious viral particles, greatly accelerated laboratory research and subsequently allowed study of the entire viral life cycle (Wakita et al., 2005). Since this initial report, molecular clones based upon genotype 1a and 2b isolates have been produced (Li et al., 2012, 2014b; Ramirez et al., 2014; Yi et al., 2006), as well as numerous intergenotypic chimaeric JFH-1 viral genomes (Gottwein et al., 2009; Li et al., 2014a; Steinmann et al., 2013); all of which are infectious in cell culture models.

Cell culture-derived HCV titres are generally measured in either focus-forming units per millilitre (FFU/mL) (reviewed in (Yi, 2010)) or through calculation of the 50% tissue culture infective dose (TCID_50_) (Reed and Muench, 1938). Unlike many viruses, HCV infection does not result in plaque formation, therefore infected cells are detected by indirect immunofluorescence using antibodies specific for virally-encoded proteins (for example, NS5A). For the majority of infectious HCV isolates developed to date, viral titres produced from the commonly used Huh-7 hepatoma cells rarely exceed 10^5^ FFU/mL. However there are extreme cases: titres of only 10^3^ FFU/mL are commonly achieved with the full-length genotype one clones, whereas the genotype 2a/2a chimaeric JC-1 virus often produces 10^6^ FFU/mL (Pietschmann et al., 2006) and is therefore an attractive alternative to the prototypic JFH-1 strain (reviewed in Vieyres and Pietschmann (2013)).

Although the measurement of viral titre in FFU remains an established method within RNA virology (Condit, 2007), in many cases advancements in technology have resulted in either automated processes or the use of surrogate markers, thereby increasing the accuracy and through put rate of the assay (for example, the virtual TCID_50_ method for HIV-1, which measures virion-associated reverse transcriptase activity (Gao et al., 2009; Marozsan et al., 2004); and the competitive virus assay for titration of bovine viral diarrhoea virus isolates (Muhsen et al., 2013)). To our knowledge, approaches such as these have not been adopted for HCV; the currently used protocol therefore remains labour-intensive, low-throughput and susceptible to significant human error during data interpretation.

In this manuscript, we describe a novel method of measuring HCV titres in infectious units (IU/mL). The experimental protocol is an adaptation of the previously-described immunofluorescence-based approaches (Lindenbach, 2009; Vieyres and Pietschmann, 2013; Yi, 2010), whilst data analysis is a wholly novel technique utilising the IncuCyte ZOOM (Essen BioScience). The IncuCyte ZOOM instrument has been utilised previously in RNA virology for detection of fluorescent reporter genes during live-cell imaging (Forrest et al., 2014; Tulloch et al., 2014), however to our knowledge this is the first application of this instrument to quantification of HCV. This method allows the accurate and precise measurement of HCV titres in a relatively high-throughput setting, thereby decreasing labour, reagent cost and human bias. The wide linear range of detection exhibited by this method also makes it highly amenable to future high-throughput screens of potential antiviral compounds.

## 2 Materials and methods

### 2.1 *In vitro* transcription of viral RNA

The DNA construct encoding the JFH-1 viral genome (pJFH-1) and a replication-defective control mutant of this plasmid (termed pJFH-1-GND, possessing a GDD > GND mutation within the RNA-dependent-RNA-polymerase active site) has been described previously (Wakita et al., 2005). The DNA construct encoding the chimaeric JC-1 virus (pJ6-JFH-1c3) has been described previously (Pietschmann et al., 2006).

DNA constructs were linearised using *Xba*I, briefly treated with mungbean nuclease to degrade 3′ overhangs (New England Biolabs) and purified by neutral phenol-chloroform extraction. Linearised DNA was used as a template for *in vitro* transcription to produce full-length HCV genomic RNA (RiboMAX Express; Promega, as per the manufacturer's instructions). Following DNAse digestion, RNA transcripts were purified by acidic phenol-chloroform extraction and quantified by absorbance at 260 nm prior to transfection into mammalian cells.

### 2.2 Mammalian cell lines

An immortalised human hepatocellular carcinoma cell line, termed Huh-7 (Nakabayashi et al., 1982) was utilised for the production and titration of all virus stocks. Huh-7 cells were maintained in Dulbecco's modified Eagle's medium (DMEM; Sigma) supplemented with 10% foetal bovine serum (FBS), 100 IU/mL penicillin, 100 ug/mL streptomycin, 25 mM HEPES and 1% (v/v) non-essential amino acids in a humidified incubator at 37 °C in 5% CO_2_.

### 2.3 Production of infectious HCV

For the production of virus stocks, 4.0 × 10^6^ Huh-7 cells were electroporated with 5 μg of viral RNA at 975 μF and 260 V for 25 ms. Cells were resuspended in complete medium and seeded into T75 flasks (Corning). Virus was harvested at 48 h post-electroporation, clarified by centrifugation (10 min, 1000 × *g*) and stored at −80 °C in 1 mL aliquots. This allowed independent experimental replicates to be performed from identical virus aliquots to assess data reproducibility.

### 2.4 Virus titration

An established method of HCV titre determination has been previously reported (Yi, 2010; Yu and Uprichard, 2010) and is generally used in a 96 well plate format (henceforth referred to as a standard titre plate). Briefly, naïve Huh-7 cells were seeded into 96 wellplates (8.0 × 10^3^ cells per well, 100 μL total volume) and allowed to adhere for 16 h. Clarified virus was serially diluted two-fold into the existing media (final volume 100 μL per well). Cells were incubated for 72 h post-infection before the detection of viral antigens by indirect immunofluorescence (Section 2.6). Virus-positive cells were counted manually and the titre (FFU/mL) was calculated from the wells of multiple virus dilutions.

### 2.5 Antiviral treatments

Huh-7 cells were electroporated with JFH-1 viral RNA as described above and 2.5 × 10^4^ cells were seeded into 96-well plates. 6 h post-electroporation, indicated concentrations of Daclatasvir (SelleckChem) or Sofosbuvir (MedChem Express) were added in duplicate with 0.25% (v/v) standard final DMSO. 72 h post-electroporation, viral supernatant was harvested and a 1:4 dilution was used to infect each well of a 96 well plate of naïve Huh-7 cells (seeded 6 h prior). 48 h post-infection, these cells were fixed with 4% paraformaldehyde and viral antigens were detected by indirect immunofluorescence. The total number of virus-positive cells therefore reflects the degree of inhibition exerted by the compound upon the original producer cells, as measured in released infectious units. 50% effective concentration (EC_50_) values were calculated using Prism (GraphPad).

### 2.6 Immunofluorescence

Infected cells were washed with phosphate-buffered saline (PBS), fixed with 4% paraformaldehyde for 15 min and extensively washed again with PBS. The fixed monolayer was permeabilised with 0.1% Triton-X100 in PBS (v/v), washed and incubated for >2 h in primary antibody raised against a viral antigen. Anti-NS5A (polyclonal sheep serum – Macdonald et al., 2003) and anti-core (Moradpour et al., 1996) (C7-50, Thermo Scientific) primary antibodies were used at 1:2000 dilutions (in PBS with 10% FBS), whereas anti-E2 (Owsianka et al., 2005) (AP33, Genentech) was used at 1:500. After further washing, cells were incubated with a fluorophore-conjugated secondary antibody (diluted 1:500 in PBS with 10% FBS) for 2 h and finally stored in PBS prior to visualisation and/or manual counting.

### 2.7 Use of the IncuCyte ZOOM

Following immunofluorescence staining for viral antigens, with either an AlexaFluor594-conjugated (“red”) or 488-conjugated (“green”) secondary antibody, microtitre plates were imaged with the IncuCyte ZOOMinstrument (Essen BioScience). The default software parameters for a 96 well plate (Corning) with a 10× objective were used for imaging. The IncuCyte software was used to calculate mean confluence from four non-overlapping bright phase images of each well. The mean number of “red” and/or “green” positive cells per image was calculated from four non-overlapping fluorescent images; this value was then extrapolated by the IncuCyte ZOOM software to calculate the total predicted number of virus-positive cells per well. Viral titres were obtained by multiplying the number of virus-positive cells per wells by the reciprocal of the corresponding dilution factor, corrected for input volume. As this method measures the absolute number of infected cells, rather than the number of foci of infected cells, the titre is represented as infectious units per mL (IU/mL).

## 3 Results

### 3.1 Optimisation of software parameters

**Fig. 1:**
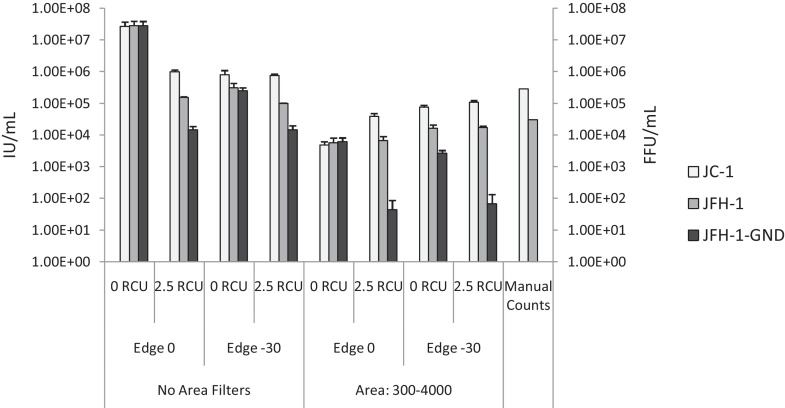
Optimisation of IncuCyte ZOOM software parameters for accurate counting of NS5A-positive cells. Images from a microtitre plate were analysed eight times, using various permutations of software parameter conditions (area filters, RCU threshold and the degree of cell-edge splits). Total “red cells per well” was calculated and extrapolated to produce viral titre in IU/mL (left axis). Data represents the mean + SEM of titres calculated from three wells across the linear range of dilutions. The combination of −30 edge split, 2.5 RCU threshold and 300–4000 μm^2^ area filters produced results most similar to those from the manual counting method, as indicated on the right axis (FFU/mL).

The standard virus titration protocol (described in Section 2.4) was used to manually determine the titres of JC-1, JFH-1 and JFH-1-GND stocks. Infected cells were fixed at 72 h post-infection and treated with a HCV NS5A-specific sheep polyclonal serum (Macdonald et al., 2003) and anti-sheep secondary antibody conjugated to the AlexaFluor 594 dye (Life Technologies). Plates were manually counted by three individuals and the average titres were calculated. Titres of 2.82 × 10^5^ (JC-1), 3.01 × 10^4^ (JFH-1) and 0.00 (JFH-1-GND) FFU/mL were determined (Fig. 1).

This plate was then imaged with the IncuCyte ZOOM. The number of “red cells per well” was calculated multiple times using a variety of software parameter permutations (Table 1). The optimal settings were determined to be a red calibrated unit (RCU) minimum threshold of 2.5, edge sensitivity of −30, and an area filter ranging from 300 to 4000 μm^2^ (Fig. 1). The results from these parameters displayed maximum correlation with the manually determined titres. Most importantly, the JFH-1-GND (replication-defective) “titre” was minimal (<100 red artefacts per ml), indicating that false positives and non-specific antibody binding events would not contribute significantly to the calculated titres of replication-competent virus. Equally, false negatives are unlikely to occur within these parameters: the RCU threshold of 2.5 prevents inclusion of cells which faintly stain for viral antigens; these are unlikely to have been infected from the initial inoculum 72 h prior to fixation, and therefore are more likely to represent new early infection events due to viral spread or background fluorescence. The exclusion of these cells would not affect accurate determination of the initial infectious viral titre.

### 3.2 Linear limitations of the dilution series

**Fig. 2:**
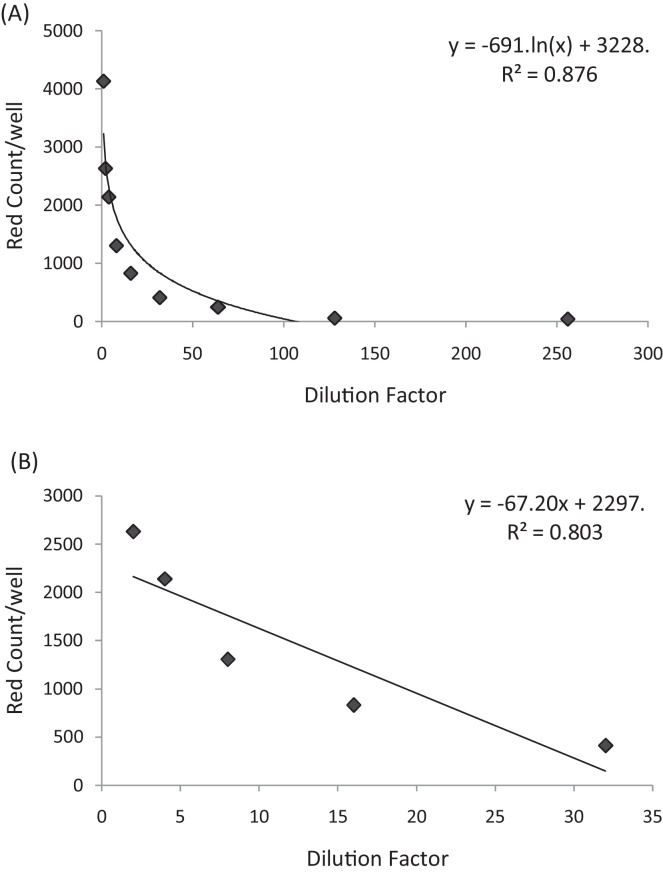
Dilutions of HCV JC-1 from 1:2^1^ and 1:2^5^ are within the linear range of the IncuCyte ZOOM. Following a two-fold titration of JC-1 virus, infected cells were fixed and stained for NS5A antigen. Total “red cells per well” was calculated for each dilution factor and a logarithmic trend was observed (A); within this data, a linear relationship was observed for dilutions 1:2^1^–1:2^5^(B).

Following establishment of the optimal software parameters, we next wished to determine the linear range of dilutions across which the IncuCyte ZOOM could produce accurate titre calculations. JC-1 virus was used to infect naïve Huh-7 cells in a two-fold dilution series. 72 h post-infection, cells were fixed and stained for NS5A as described above. As expected, the number of virus-positive cells *versus* the dilution factor displayed a logarithmic trend (Fig. 2A), and a linear range was observed for dilutions between 1:2^1^ and 1:2^5^(Fig. 2B). For this particular virus preparation, this equates to an average titre calculation of 1.14 × 10^5^ IU/mL. Realistically an accurate manual count of foci and/or virus-positive cells can only be obtained for one or two wells in a serial dilution series before the human error will impact upon the significance; thus this linear range is a significant improvement on the previously-used assay.

### 3.3 Optimisation of the titration protocol

The traditional method of HCV titration involved pre-seeding the cells 16–24 h before infection, which was then allowed to proceed for 48–72 h before fixation (Lindenbach, 2009; Yi, 2010; Yu and Uprichard, 2010). Although a 72 h infection period may allow a degree of viral spread (thereby obscuring accurate measurement of the initial infectious inoculum) this extended time period was required to ensure sufficient levels of viral protein per infected cell; hence the strength of antibody staining was sufficiently high for visualisation of infected foci by eye.

**Fig. 3:**
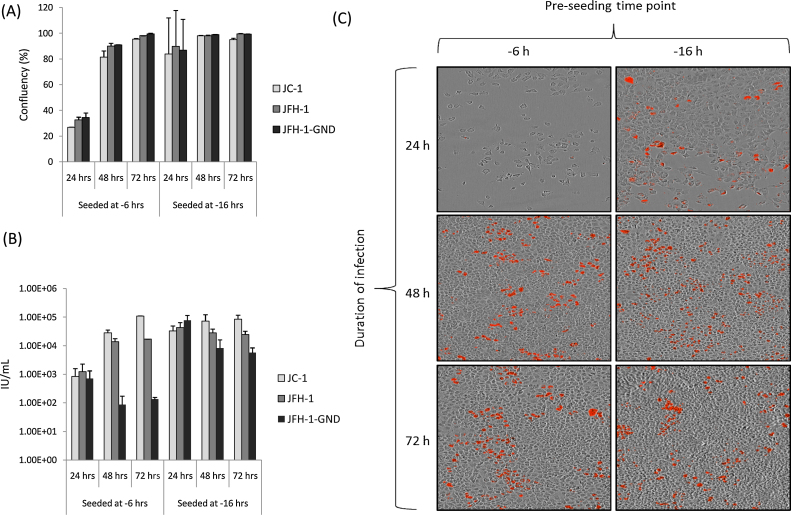
Optimisation of pre-seeding and infection time periods. Cells were seeded either 6 or 16 h prior to infection, which was then allowed to proceed for 24, 48 or 72 h before fixation. The optimal confluency was obtained when cells were seeded at −6 h and fixed at 48 h post-infection (A); this correlated to the lowest background count of red cells per well in the GND controls (B) and is therefore the optimal time frame. Data represents the mean + SEM of titres calculated from three independently infected titre plates. (C) Representative blended images (bright phase-red fluorescence) of Huh-7 monolayers seeded and infected with JFH-1 at the indicated time points. The optimal confluency and most easily identifiable fluorescent cells are visible in the 6 h pre-seeding, 48 h infection duration panel (central left).

We therefore investigated the optimal pre-seeding and infection time conditions for use with the IncuCyte ZOOM. Identical cell numbers were maintained from previous assays (100 μL of 8.0 × 10^3^ cells/mL per well). Cells were seeded 6 h or 16 h prior to infection, which was then allowed to proceed for 24, 48 or 72 h before monolayers were fixed and stained for the NS5A viral antigen. Plates were imaged and analysed with the optimised parameters described in Fig. 1. The optimal conditions, producing low levels of false positives in the GND controls and a confluence of approximately 87%, were the combination of pre-seeding 6 h before infection and fixation at 48 h (Fig. 3). This represents a significant increase in the rapidity of the assay compared to the previous protocol.

### 3.4 Assay flexibility

**Fig. 4:**
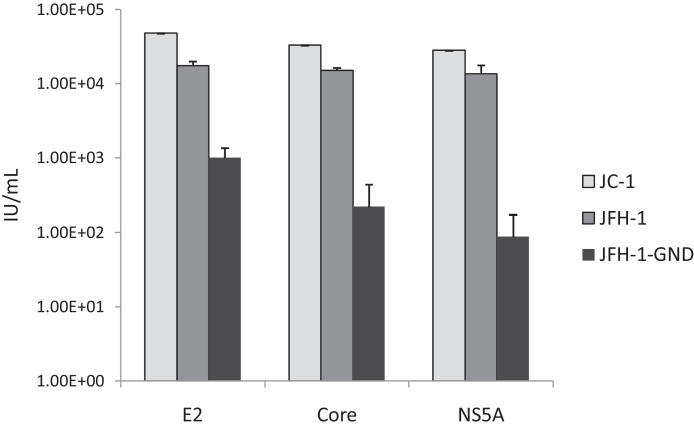
A range of HCV-specific commercial antibodies are suitable for use in the IncuCyte ZOOM-adapted titration protocol. Following infection and fixation, cells were stained for HCV E2, core or NS5A proteins and imaged and analysed using described optimised parameters. Similar titre values for JFH-1 and JC-1 isolates were obtained for all antibodies investigated. Data represents the mean + SEM of titres calculated from three independently infected titre plates.

For the majority of experiments we utilised an in-house sheep polyclonal serum raised against HCV NS5A (Macdonald et al., 2003), in combination with an anti-sheep secondary antibody labelled with the AlexaFluor 594 dye (Life Technologies). However it is important that this assay may be adapted to utilise a range of commercially available primary and secondary antibodies. A virus titration assay was performed according to the optimised protocol (cells seeded 6 h prior to infection, and fixed 48 h post-infection). Fixed infected cells were then stained with commercially-available E2-specific (Owsianka et al., 2005) (AP33, Genentech) or core-specific mouse monoclonal antibodies (Moradpour et al., 1996) (C7-50, Thermo Scientific) (Fig. 4). Both of these monoclonal antibodies recognise linear epitopes that are conserved across HCV genotypes and as such would be applicable to other, non-JFH-1-based virus titration assays. Anti-mouse secondary antibodies labelled with either the 488 (for E2 staining) or 594 dye (for core staining) were used. It must be noted that the 488-labelled secondary antibody displayed higher background staining and therefore the minimal green calibrated unit (GCU) threshold was increased to 4.5. Plates were imaged and analysed according to the optimised software parameters. Similar titres were obtained for both JFH-1 and JC-1 for all antibodies tested, although higher background staining of the GND control wells was observed for the E2-stained samples (Fig. 4). These results demonstrate that this assay is amenable to a range of primary and secondary antibodies and is therefore readily accessible to other laboratories.

### 3.5 Validation of antiviral compound screening

**Fig. 5:**
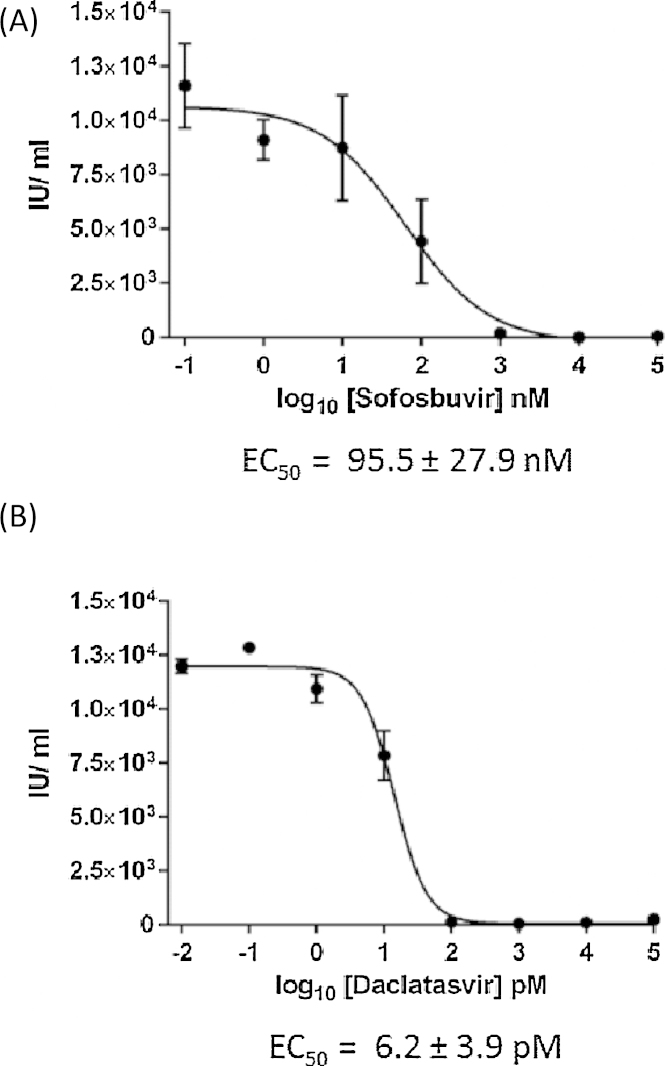
The IncuCyte ZOOM titration assay predicts accurate EC_50_ values for known antivirals and is therefore applicable for the identification of novel compounds. Electroporated cells were treated with a range of concentrations of anti-HCV antiviral compounds, Sofosbuvir (A) or Daclatasvir (B). Virus was harvested at 72 h and a 1:2^2^ dilution was used to infect naïve Huh-7 cells. 48 h post-infection, cells were fixed and stained for NS5A. Calculated titres were plotted and used to predict EC_50_ values (±SEM) for each compound. Graphed data are representative of three independent experimental replicates.

The increased-throughput measure of IU/mL for this assay may be particularly useful in the study of antivirals that target multiple stages of the virus lifecycle (McGivern et al., 2014). Thus, an adapted protocol was used to calculate EC_50_ values for the direct acting antivirals Daclatasvir and Sofosbuvir, both of which are in clinical use for HCV therapy. Cells electroporated with JFH-1 RNA were incubated with a range of inhibitor concentrations, before naïve cells were infected using previously optimised dilutions within the accurate linear range (in this case, 1:2^2^ as displayed in Fig. 3). Infected cells were stained for NS5A, imaged and analysed as described. The extrapolated IU/mL was then plotted to calculate the EC_50_ of each compound (Fig. 5). The values reported (95.5 ± 27.9 nM for Sofosbuvir and 6.2 ± 3.9 pM for Daclatasvir) are within the published range for each antiviral compound, validating this approach for the screening of novel compounds in the future.

## 4 Discussion

In this paper, we have described the optimisation of a novel approach for analysing HCV infectious titres. The previously published protocols involve a 4-day assay followed by lengthy manual counting of infected cells; in contrast, our method requires only 3 days of cell incubation followed by fully automated data analysis. This represents a significant increase in processivity, as well as improved accuracy, precision and reproducibility. The linear range of detection that this method exhibits also means that dilution to an endpoint is not essential, in contrast to both the focus-forming unit and TCID_50_ methods. Therefore a reduced number of viral dilutions are required for each sample, significantly reducing the reagent cost per assay.

Alternatives to HCV titrations have been investigated previously but all possess significant caveats; for example, antigen-capture based techniques, such as core protein ELISAs, are high-throughput but neither differentiate between defective particles and infectious virions, nor antigen released from dead cells. Thus, such measurements are poor surrogates for infectious titre assays. Other assays rely upon the expression of reporter genes inserted into the viral genome; for example, luciferase or green fluorescent protein. However the quantification of a reporter gene relies upon assumption of a linear relationship between reporter expression levels and infectious units; a controversial assumption which would require validation for each novel clone produced. Equally concerning is the fact that insertion of a reporter gene tends to decrease viral fitness (Vieyres and Pietschmann, 2013). As research is focussed increasingly upon the development of novel, non-JFH-1-based infectious clones, which are more relevant to clinical isolates but tend to display low infectious titres, insertion of a reporter is unlikely to represent a feasible option. To circumvent this, reporter-based cell lines have been described which facilitate the study of native virus (Iro et al., 2009; Jones et al., 2010). These offer an attractive method for the quantification of HCV titres that is genotype independent, but require the establishment of an appropriate cell line expressing the reporter of interest. For example, Jones et al. (2010) describe a method in which a fluorescent reporter (either GFP or mCherry), is fused to both a nuclear localisation signal and the C-terminal 78 residues of the mitochondrial antiviral signalling protein (MAVS). This region contains both a mitochondrial targeting sequence and a cleavage site for NS3/4A. Upon infection, NS3/4A cleaves this reporter and the fluorescent protein is released from the mitochondria and translocates to the cell nucleus. The image processing required to distinguish between uninfected and infected cells using this approach is thus substantially more complex than that described here which simply enumerates positive cells identified by immunostaining. Our method may potentially be applicable to multiple infectious HCV isolates, given the successful use of various primary antibodies targeting conserved epitopes. To the best of our knowledge, this is the first automated, high-throughput method of accurately analysing viral infectious titres, which does not rely upon expression of a reporter gene.

This method has applications for multiple areas of HCV research, as most require accurate measurements of viral release and infectivity. We predict the main use to be in the identification and validation of novel antiviral compounds. The reported EC_50_ for Sofosbuvir is 558 nM for a genotype 2a/2a chimaeric JFH-1-based infectious virus (Ramirez et al., 2014); in comparison, Daclatasvir exerts an EC_50_ of 28 pM (Gao et al., 2010). Therefore our reported calculations are within the expected range for infectious virus for both compounds (Fig. 5); this validates the use of our assay for the accurate calculation of EC_50_ values. Equally, the measurement of neutralising antibody titres would benefit significantly from an adaptation of our protocol. A reduction in foci (caused by infectious virus following incubation with patient sera) is already commonly used as a method of assessing neutralising potential (Fournier et al., 2007); the application of our optimised protocol would significantly enhance the throughput of these studies.

In conclusion, we have developed and optimised a novel method of measuring and analysing HCV infectious titres, which requires the use of the IncuCyte ZOOM instrument and associated software. This assay will prove useful for all basic research involving novel infectious HCV isolates and the identification of novel antivirals targeting multiple stages of the viral life cycle.

## Figures and Tables

**Table 1:** Definitions of parameters used within the IncuCyte ZOOM software.

Parameter	Function
RCU threshold	A fixed threshold level (in red calibrated fluorescence units) is used across the image, so all objects below this level are classified as background and not included in calculations
Edge sensitivity	Edge split sensitivity determines whether closely-spaced objects are separated, according to the weak signal points between them. A larger edge sensitivity number (*i.e.*, 0 compared to −30) results in more splits, producing a higher count
Area filter	This function eliminates fluorescent objects that are outside the chosen size (in μm^2^)
